# Simultaneous Determination of Decursin, Decursinol Angelate, Nodakenin, and Decursinol of *Angelica gigas* Nakai in Human Plasma by UHPLC-MS/MS: Application to Pharmacokinetic Study

**DOI:** 10.3390/molecules23051019

**Published:** 2018-04-26

**Authors:** Sook-Jin Kim, Se-Mi Ko, Eun-Jeong Choi, Seong-Ho Ham, Young-Dal Kwon, Yong-Bok Lee, Hea-Young Cho

**Affiliations:** 1 College of Pharmacy, CHA University, 335 Pangyo-ro, Bundang-gu, Seongnam-si, Gyeonggi-do 13488, Korea; supia925@gmail.com (S.-J.K.); 92kosemiya@gmail.com (S.-M.K.); gonru@naver.com (E.-J.C.); 2 National Development Institute of Korean Medicine, 288 Udeuraendeu-gil, Anyang-myeon, Jangheung-gun, Jeollanam-do 59338, Korea; phd_ham@nikom.or.kr; 3 Department of Oriental Rehabilitation Medicine, Wonkwang University Gwangju Medical Center, 1140-23 Hoejae-ro, Nam-gu, Gwangju 61729, Korea; kwonyd@wonkwang.ac.kr; 4 College of Pharmacy, Chonnam National University, 77 Yongbong-ro, Buk-Gu, Gwangju 61186, Korea; leeyb@chonnam.ac.kr

**Keywords:** UHPLC-MS/MS, decursin, decursinol angelate, nodakenin, decursinol

## Abstract

Coumarins in Cham-dang-gwi, the dried root of *Angelica gigas* Nakai (AGN), possess pharmacological effects on anemia, pain, infection, and articular rheumatism. The AGN root containes decursin (D), decursinol angelate (DA), nodakenin, and decursinol (DOH), a major metabolite of D and DA. The aim of this study was to develop a simultaneous determination method for these four coumarins in human plasma using ultra high performance liquid chromatography-tandem mass spectrometry (UHPLC-MS/MS). Chromatographic separation was performed on dual columns (Kinetex^®^ C_18_ column and Capcell core C_18_ column) with mobile phase consisting of water and acetonitrile at a flow rate of 0.3 mL/min using gradient elution. Multiple reaction monitoring was operated in positive ion mode with precursors to product ion transition values of *m*/*z* 328.9→228.8, 328.9→228.9, 409.4→248.8, and 246.8→212.9 to measure D, DA, nodakenin, and DOH, respectively. Linear calibration curves were fitted over concentration range of 0.05–50 ng/mL for these four components, with correlation coefficient greater than 0.995. Inter- and intra-day accuracies were between 90.60% and 108.24%. These precisions were within 11.19% for all components. The established method was then applied to a pharmacokinetic study for the four coumarins after usual dosing in Korean subjects.

## 1. Introduction

**Figure 1:**
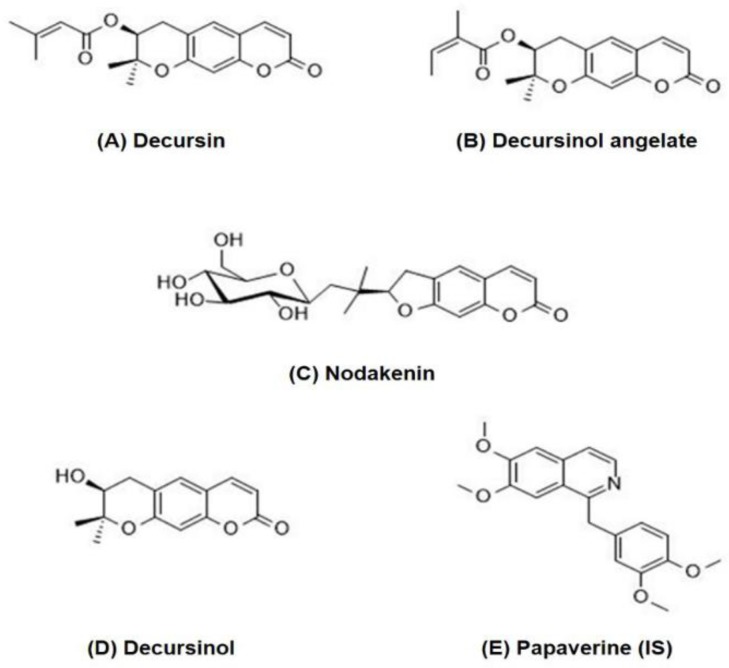
Structures of the four coumarins and papaverine as an IS. (**A**) decursin, (**B**) decursinol angelate, (**C**) nodakenin, (**D**) decursinol, and (**E**) papaverine (IS).

The dried root of *Angelica gigas* Nakai (AGN) (Cham-dang-gwi in Korea) is a traditional medicine widely used to treat anemia, pain, infection, and articular rheumatism in Korea and other Asian countries [1,2]. Coumarins have been found to be predominant constituents of Cham-dang-gwi extract [2]. Coumarins in AGN are composed of decursin (D), decursinol angelate (DA), nodakenin, and decursinol (DOH) as a major metabolite of D and DA (Figure 1). As major active components of AGN, they possess pharmacological and biochemical activities both in vitro and in vivo, including anti-cancer [3,4] and neuroprotective activities [5]. D and DA have shown significant antibacterial activities [6]. Nodakenin has anti-imflammatory effects [7]. Nodakenin can also increase cognitive function and adult hippocampal neurogenesis in mice [8]. It can palliate scopolamine-induced memory and cognitive impairment due to its inhibition on acetylcholinesterase activity [9]. In addition, D and DA are rapidly metabolized to DOH in liver microsomes of humans and rodents [6]. As an active metabolite, DOH is well-known for its antinociceptive [10], analgesic [11], and sepsis-preventing effects [12].

Despite these pharmacological activities, pharmacokinetic (PK) studies for major components of AGN are limited. Several PK studies of AGN in mice [13], rats [14,15,16,17,18,19,20,21,22], and humans [23] have been published. Most of them have characterized PKs of one or two components of courmarins from extracted AGN or shown PKs of combined D and DA. One publication has reported PKs for four coumarins of D, DA, nodakenin, and DOH [14]. However, only combined D and DA could be evaluated in that study because their analytical methods could not separate isomeric peaks of D and DA. Therefore, it is necessary to develop a new method that can separate peaks of D and DA.

During the past few decades, many analytical methods using various quantitative analysis instruments—including HPLC-UV [13,14,15,16,17,20], LC-MS/MS [18,19], and UHPLC-MS/MS [23]—have been reported for coumarins from AGN root. Most of these publications have suggested analytical methods for one or two components of coumarins in rat plasma. Simultaneous quantitation of D, DA, and DOH has been reported in mouse plasma with lower limit of quantification (LLOQ) of 250 ng/mL by using HPLC-UV [13] and in rat plasma with LLOQ of 0.2 ng/mL using LC-MS/MS [21]. The highest sensitivity at LLOQ of 0.1–1 ng/mL for detecting D, DA, and DOH in human plasma has been reported by using UPLC-MS/MS with solid supported liquid extraction (SLE) [23]. Analytical techniques of nodakenin in rat plasma have been reported with LLOQ of 100 ng/mL [20] and 250 ng/mL [16] using HPLC-UV and 2 ng/mL using LC-MS/MS [19]. However, no study has reported simultaneous quantification of the four major components of AGN (i.e., D, DA, nodakenin, and DOH) in human plasma. Although Hwang et al. [14] have reported an analytical method for detecting four coumarins with LLOQ of 50–10 ng/mL using HPLC-UV, they could not separate isomeric peaks of D and DA. Only two studies have separated isomers of D and DA using a single column with a long run time of 30 min [21] and dual columns having different diameters and lengths at a relative short run time of 9 min [23]. Therefore, there is a need to develop new cost- and time-saving simultaneous quantification methods for four coumarins from the AGN.

The objective of the present study was to develop a sensitive, selective, and validated analytical method using UHPLC-MS/MS for simultaneous determination of D, DA, nodakenin, and DOH in human plasma. The established method was then used to investigate PKs of these four coumarins after oral administration of AGN root extract powder (0.055 mg as D, 0.184 mg as DA, and 1.095 mg as nodakenin) to Korean subjects.

## 2. Results and Discussion

### 2.1. Method Development

**Figure 2:**
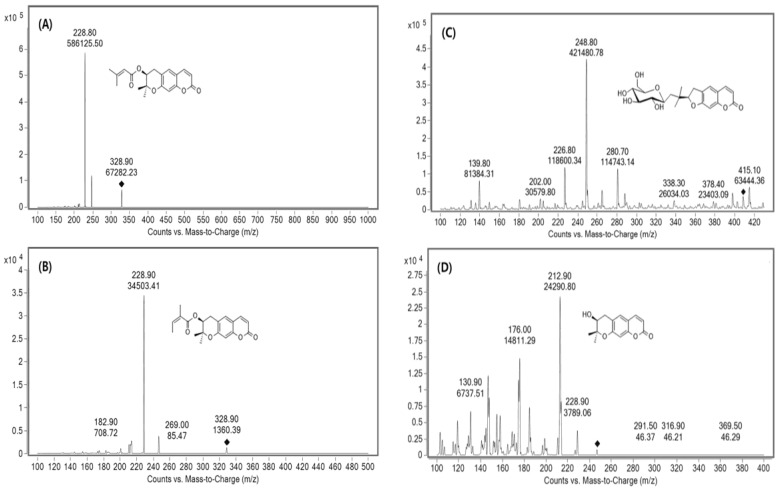
Full scan product ions of precursor ions of (**A**) decursin (328.8→228.8), (**B**) decursinol angelate (328.9→228.9), (**C**) nodakenin (409.4→248.8), and (**D**) decursinol (246.8→212.9).

MS/MS spectra were tested in each positive and negative ion ESI mode. The positive mode was adopted because it had better sensitivity than negative mode. The four coumarins were identified based on retention time and precursor-to-product ion pair mass ratio. Product ion mass spectra of D, DA, nodakenin, DOH, and IS were obtained after injecting individual standard solution into the mass spectrometer as shown in Figure 2.

UHPLC conditions including columns, column temperature, mobile phase system, and flow rate were investigated to optimize the sensitivity, retention time, and resolution. In previous literature, water/acetonitrile [13,14,16,21,23] or water/acetonitrile containing formic acid [15,18,19] was used as the mobile phase for the detection of coumarins. Therefore, we tested these two conditions. When mobile phase containing 0.1% formic acid was used, results were unsatisfactory due to poor sensitivity and resolution. Above all, when 0.1% formic acid was used, a peak of D and DA was not separated at all. A mobile phase consisting of water and acetonitrile showed better resolution and high intensity. However, the chromatogram showed double peak for D and DA. Therefore, water/acetonitrile was preferentially chosen as the mobile phase to achieve better peak shape, high sensitivity, stable base line, and gradient elution with satisfying retention time and interference peaks.

**Figure 3:**
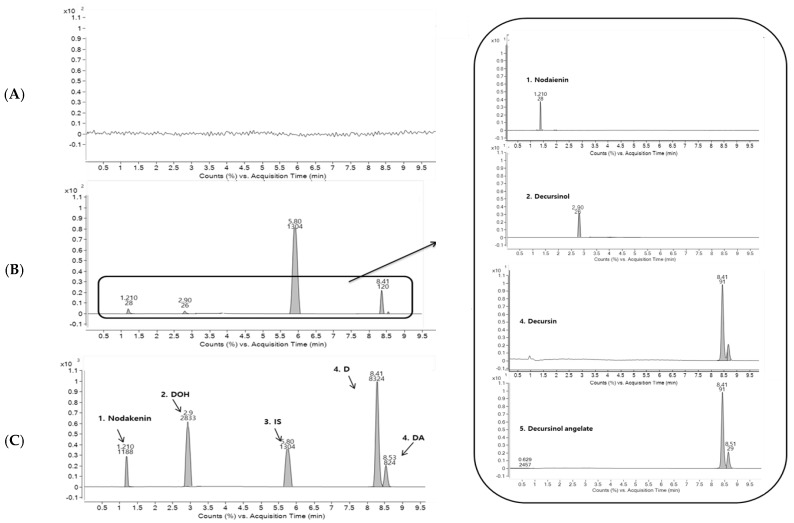
Representative MRM chromatograms of AGN root extract in human plasma samples (1, nodakenin; 2, decursinol; 3, IS; 4, decursin; and 5, decursinol angelate). (**A**) blank human plasma, (**B**) human plasma spiked with the four coumarins at LLOQ of 0.05 ng/mL and IS (10 ng/mL), (**C**) human plasma taken at 1 h after the usual oral dose administration of 4.6 g of AGN root extract powder containing 0.055 mg of D, 0.184 mg of DA, and 1.095 mg of nodakenin.

To separate isomeric peaks of D and DA, we used various columns with different sizes, and packing technologies based on octadecyl-silica including Kinetex C_18_ column (100 × 3.1 mm, 2.6 μm particle size, Phenomenex, Torrance, CA, USA), Capcell core C_18_ column (50 × 2.1 mm, 2.7 μm particle size, Shiseido, Tokyo, Japan), HALO-2 C_18_ column (50 × 2.1 mm, 2 μm particle size, Advanced materials technology, Wilmington, DE, USA), and Luna Omega C_18_ column (100 × 2.1 mm, 1.6 μm particle size, Phenomenex). These columns had the same packing material (octadecyl-silica) but different characteristics depending on their applications. Kinetex C_18_ column showed increased retention time for polar compounds. Capcell core C_18_ column had good stability at any pH conditions due to its polymer coating. HALO-2 C_18_ column had excellent performance for ionizable compounds. Luna Omega C_18_ column was focused on hydrophobic retention. When a single column was used, all columns could not separate isomer peaks of D and DA despite various gradient conditions were tried. Therefore, we connected two columns (Kinetex C_18_ and Capcell core C_18_ columns) to separate peaks of D and DA based on the study of Zhang et al. [23]. Isomeric peaks of D and DA were completely separated. We determined the two compounds through the experimental results which were obtained after injecting individual standard solution of D and DA. Optimized chromatograms for all four coumarins are shown in Figure 3. As a result, the use of dual columns of Kinetex C_18_ column (100 × 3.1 mm, 2.6 μm particle size, Phenomenex) and Capcell core C_18_ column (50 × 2.1 mm, 2.7 μm particle size, Shiseido) showed better resolution, peak shape, and separation than using a single column. The developed method reduced the analytical time about three-fold greater compared to the time using a single column (30 min) [21]. In addition, the flow rate of mobile phase, column temperature, and injection volume were examined considering sensitivity and column pressure. Finally, optimized conditions were: injection volume, 5 μL; flow rate, 0.3 mL/min; column temperature, room temperature.

For sample preparation, protein precipitation (PP) and liquid–liquid extraction (LLE) methods were tried. We compared these two procedures (PP with methanol and acetonitrile, LLE with ethyl acetate and methyl *tert*-butyl ether) to determine the better sample preparation method. LLE was not selected due to lower recovery than PP. When methanol was used in comparison with other solvents, the largest amount of analyte was extracted. To obtain high sensitivity, we concentrated the supernatant after PP with methanol. These methods showed minimum interference with high recovery. In the previous literature, Zhang et al. [23] reported the highest sensitivity (LLOQ of 0.1–1 ng/mL for D, DA, and DOH) by using commercial kit such as SLE for sample preparation. We developed more simple, sensitive (at least 2 times), and cost-effective method using PP than theirs.

### 2.2. Method Validation

#### 2.2.1. Specificity

Representative chromatograms of blank human plasma (A), human plasma spiked with four coumarins at LLOQ of 0.05 ng/mL and IS (10 ng/mL) (B), and human plasma taken at 1 h after oral administration of AGN root extract powder (C) are shown in Figure 3. For, blank plasma, there were no significant chromatographic interferences for analytes or IS around their retention times. Retention times of D, DA, nodakenin, DOH, and IS were 8.50, 8.73, 1.58, 3.67, and 5.20 min, respectively. The signal-to-noise ratio of LLOQ and was greater than 10:1, which is 23.7:1 for D, 32.8:1 for DA, 11.1:1 for nodakenin, and 12.8:1 for DOH, respectively.

#### 2.2.2. Calibration Curves and LLOQ

Calibration curves for the four coumarins in human plasma showed good linearity over concentration range of 0.05 to 50 ng/mL for D, DA, nodakenin, and DOH with a correlation coefficient (*r*) of more than 0.995. Typical linear regression equations were: *y* = (0.6289 ± 0.0206)*x* + (0.3339 ± 0.0423) for D, *y* = (0.3342 ± 0.0227)*x* + (0.0033 ± 0.0011) for DA, *y* = (0.1534 ± 0.0433)*x* + (0.0104 ± 0.0133) for nodakenin, and *y* = (0.2542 ± 0.0452)*x* + (0.051 ± 0.0028) for DOH. LLOQs of D, DA, nodakenin, and DOH were all at 0.05 ng/mL. Sufficiently low LLOQ values for the PK study after oral administration of AGN root extract powder in humans could be obtained through optimization of the UHPLC-MS/MS method.

To the best of our knowledge, the most sensitive analytical method for coumarins using UPLC-MS/MS system has an LLOQ of 0.1 ng/mL for D and DA and 1 ng/mL for DOH in human plasma [23]. However, that method used SLE for sample preparation. It did not include nodakenin. An LLOQ of 0.2 ng/mL for D, DA, and DOH using LC-MS/MS with LLE has also been reported previously [21]. However, its LLOQ value is at least four-times higher than that in our study. Other methods have shown lower sensitivity, with LLOQs ranging from 50 to 250 ng/mL for combined D and DA [13,14,17], 2 to 100 ng/mL for nodakenin [16,19,20], and 39 to 200 ng/mL for DOH [13,14,17,18]. In comparison, our simultaneous analytical method was the most sensitive, simple, and cost-effective for determining the four coumarins in plasma (LLOQ of 0.05 ng/mL for all components with the PP method using 0.1 mL plasma). In addition, D and DA could be individually analyzed due to separation of the isomeric peak. Therefore, for the first time, we developed a simultaneous analysis method for D, DA, nodakenin, and DOH of AGN in human plasma.

#### 2.2.3. Precision and Accuracy

Results of intra- and inter-batch precision and accuracy of D, DA, nodakenin, and DOH are summarized in Table 1. The intra-batch accuracy ranged from 95.64% to 102.67% for D, 99.25% to 105.70% for DA, 90.67% to 99.06% for nodakenin, and 90.60% to 108.24% for DOH. The precision (coefficient of variation, CV) was less than 6.78% for D, 8.89% for DA, 11.19% for nodakenin, and 9.09% for DOH. Inter-batch accuracy for four coumarins ranged from 94.16% to 107.31% for D, from 97.36% to 103.74% for DA, from 93.56% to 97.71% for nodakenin, and from 96.13% to 107.04% for DOH. The precision (CV) was within 9.44% for D, 5.86% for DA, 8.50% for nodakenin, and 6.17% for DOH. All these values were within the acceptable criteria (±15% for QC samples and ±20% for LLOQ). Therefore, this method showed suitable precision, accuracy, and reproducibility.

#### 2.2.4. Recovery and Matrix Effects

Extraction recoveries for analytes and IS from human plasma were 90.4 ± 5.3% for D, 89.1 ± 8.1% for DA, 94.2 ± 2.0% for nodakenin, 82.7 ± 8.1% for DOH, and 84.2 ± 11.4% for IS. Matrix effects were 82.8 ± 9.4% for D, 92.6 ± 5.2% for DA, 94.1 ± 5.7% for nodakenin, and 85.2 ± 1.3% for DOH. Precision values of D, DA, nodakenin, and DOH were 12.2, 6.0, 11.5, and 1.7%, respectively, all of which were within acceptable criteria. Therefore, this simple PP method is suitable for sample preparation to determine the four coumarins in human plasma.

#### 2.2.5. Stability

The stability of each of the four courmarins was evaluated under various storage conditions using standards and quality control (QC) samples with low and high concentrations. Results are summarized in Table 2. The four coumarins showed no significant degradation in human plasma under any conditions tested, including storage at room temperature for 8 h, after three freeze-thaw cycles, storage in auto sampler after sample preparation (10 °C) for 24 h, or under storage condition of −80 °C for a month. The stability mean values were in the range of 96.54–108.94 for D, 99.35–111.85 for DA, 101.43–111.62 for nodakenin, and 87.35–106.93 for DOH. Therefore, these four coumarins were stable under different storage conditions during the study period.

#### 2.2.6. Incurred Sample Reanalysis (ISR)

ISR assay was performed by reanalyzing 17 clinical samples. The variability (12.14) between mean value of the initial analysis and that of the reanalysis was within ±15%. Reanalysis values for two-thirds of all samples were within 20% of their initial values.

### 2.3. Contents of D, DA, and Nodakenin in Roots of AGN

Contents of D (C_19_H_20_O_5_: 328.36 g/mol), DA (C_19_H_20_O_5_: 328.36 g/mol), and nodakenin (C_20_H_24_O_9_: 408.40 g/mol) in 0.5032 g of the AGN root extract powder were calculated to be: 0.006 mg of D, 0.0201 mg of DA, and 0.1198 mg of nodakenin.

### 2.4. PK Study

**Figure 4:**
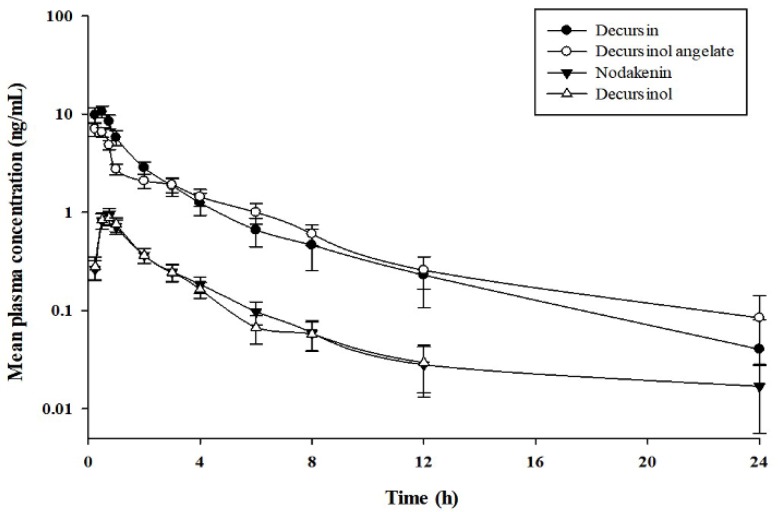
Mean plasma concentration–time profiles of four coumarins after oral administration of AGN root extract powder (4.6 g) in humans (Mean ± SE, *n* = 10).

The validated simultaneous UHPLC-MS/MS analytical method was applied to a PK study of D, DA, nodakenin, and DOH after a single oral administration of 4.6 g of AGN root extract powder (0.055 mg as D, 00.184 mg as DA, and 1.095 mg as nodakenin) to 10 healthy Korean subjects. The administered dose was a usual dose of AGN root extract powder approved in Ministry of Food and Drug Safety (MFDS). Mean plasma concentration–time curves of the four compounds in humans are shown in Figure 4.

PK parameters of the four coumarins were analyzed by non-compartmental method. Results are listed in Table 3. Values of the elimination half-life (t_1/2_) for D, DA, and DOH were similar to each other (3.03, 4.04, and 2.62 h, respectively). They were shorter than t_1/2_ of nodakenin (6.28 h). Time to reach the maximum plasma concentration (T_max_) values for D and DA (0.44 and 0.31 h, respectively) were similar to each other. T_max_ values for nodakenin and DOH (0.67 and 0.64 h, respectively) were also similar to each other.

PK study of the four coumarins has been reported in several studies. However, presented PK parameters are inconsistent with each other [14,18,21,23]. Of these studies, only one paper has reported a PK study for D, DA, and DOH after oral administration in humans (male, *n* = 10) for AGN-based dietary supplement Cogni Q (119 mg D and 77 mg DA from four vegicaps) [23]. Their t_1/2_ was longer than ours: about 5.84 times longer for D (17.7 ± 7.8 h), 3.7 times for DA (14.9 ± 2.8 h), and 2.5 times for DOH (6.6 ± 1.9 h). Such discrepancies in PK parameters between that study and the current study might be due to differences in dosage and the influence of excipients from different formulations. PKs of nodakenin in humans have not been reported previously.

In summary, simultaneous quantification method of D, DA, nodakenin, and DOH in human plasma was fully applied to a PK study after oral administration of AGN extract power (containing 0.055 mg of D, 0.184 mg of DA, and 1.095 mg of nodakenin). This study is the first one that reports PKs of the four coumarins after administering AGN root extract powder to humans.

## 3. Materials and Methods

### 3.1. Chemicals and Reagents

D (purity ≥98%) and papaverine (internal standard, IS) (purity ≥98%) were obtained from Sigma-Aldrich (St. Louis, MO, USA). Nodakenin (purity ≥98%), DA (purity ≥98%), and DOH (purity ≥98%) were purchased from Chengdu Biopurify Phytochemicals Ltd. Korea (Seoul, Korea). AGN root extract powder was obtained from Hanzung Pharmaceutical Co., Ltd. (Daejeon, Korea). Acetonitrile and methanol of HPLC grade were purchased from J.T. Baker (Phillipsburg, NJ, USA). Distilled water (18.2 MΩ) was prepared with an ElgaPurelab option-Q system (ElgaLabwater, Marlow, UK) for this study.

### 3.2. Instrumentation and Chromatographic Conditions

Simultaneous quantitative detection method for D, DA, nodakenin, and DOH in human plasma was developed by using an Agilent 1260 Series UHPLC system coupled with an Agilent 6495 mass spectrometer (Agilent Technologies, Palo Alto, CA, USA). Chromatographic separation was optimized on dual columns of Kinetex C_18_ column (100 × 3.1 mm, 2.6 μm particle size, Phenomenex, Torrance, CA, USA) and Capcell core C_18_ column (50 × 2.1 mm, 2.7 μm particle size, Shiseido, Tokyo, Japan) at room temperature. Mobile phase consisted of water (solvent A) and acetonitrile (solvent B) using a gradient elution program as follows: 0.0–2.0 min (25% B), 2.0–3.0 min (25–50% B), 3.0–8.0 min (50% B), 8.0–9.0 min (50–25% B), and 9.0–10.0 min (25% B). Flow rate was set at 0.3 mL/min and the injection volume was 5 μL. Mass spectrometry with an electrospray ionization source was operated in positive ion mode with, multiple reaction monitoring (MRM) transitions (*m/z* 328.9→228.8 for D, *m/z* 328.9→228.9 for DA, *m/z* 409.4→248.8 for nodakenin, *m/z* 246.8→212.9 for DOH, and *m/z* 339.8→323.8 for an IS). MS parameters for quantification of these four coumarins were optimized at gas temperature of 200 °C, gas flow of 15 L/min, sheath gas heater at 250 °C, sheath gas flow of 11 L/min, nebulizer pressure of 7 psi, and capillary voltage of 3000 V. Each collision energy of D, DA, nodakenin, DOH, and IS was set at 21, 25, 13, 37, and 33 eV, respectively (Table 4). Data acquisition and analysis were processed using Agilent Mass Hunter software (Agilent Technologies, Palo Alto, CA, USA).

### 3.3. Preparation of Standards and QC Samples

Standard stock solutions of D, DA, nodakenin, DOH, and IS were individually prepared in methanol at a concentration of 1 mg/mL and stored in a refrigerator (−20 °C). Standard working solutions of these four coumarins were prepared by diluting the standard stock solution with 50% methanol in water (50:50, *v*/*v*) to a series of appropriate concentrations (0.5, 1, 5, 10, 50, 100, and 500 ng/mL). The IS solution was diluted in 50% methanol in water to a final concentration of 10 ng/mL. Samples used for generating the standard calibration curve and QC were prepared by spiking 10 µL of the standard working solution in 90 µL of blank human plasma. For standard calibration curves, concentration ranged from 0.05 to 50 ng/mL for all components. QC samples for D, DA, nodakenin, and DOH were prepared at 0.05, 0.15, 8.0, and 40 ng/mL in the same manner. All calibration curve and QC samples were conducted on the day of analysis.

### 3.4. Sample Preparation

The four coumarins were extracted from human plasma samples by PP with methanol. A 100 µL of human sample was mixed with 10 µL of IS solution (10 ng/mL). The mixture was added to 900 µL of methanol, vortexed for 3 min using a vortex mixer, and centrifuged at 10,000× *g* for 5 min at room temperature. A 700 µL of the supernatant was moved to a clean tube and evaporated under nitrogen at 50 °C. The extract was reconstituted with 100 µL of acetonitrile-water (1:1, *v*/*v*) and vortexed for 1 min. After centrifugation at 10,000× *g* for 5 min, 10 µL of the supernatant was injected into the UHPLC-MS/MS system.

### 3.5. Method Validation

For simultaneous quantitative analysis, optimized UHPLC-MS/MS method was validated based on specificity, linearity, precision, accuracy, recovery, matrix effect, and stability for the four coumarins. Validation of the method was carried out in accordance with the Guidance for Industry: Bioanalytical Method Validation [24].

#### 3.5.1. Specificity

Specificity was investigated by comparing interferences of endogenous compounds using screening analysis of blank samples from six different sources and LLOQ samples. Acceptable criteria of LLOQ are within 80–120% of accuracy. The presence of components in plasma samples should not influence the measurement of these four analytes.

#### 3.5.2. Linearity and LLOQ

All calibration curves for the four coumarins were validated with a series of standard samples in concentration range of 0.05–50 ng/mL in human plasma. Linearity was determined by correlation coefficient (*r*) of each calibration curve constructed by plotting peak area ratio (*y*) of each analyte to the IS versus nominal plasma concentration (*x*) using weighted (1/*x^2^*) least square linear regression. Sensitivity was expressed as LLOQ (the lowest concentration of standard samples giving a signal-to-noise ratio of at least 10:1), within acceptable accuracy between 80 and 120% of the theoretical value and precision less than 20% CV.

#### 3.5.3. Accuracy and Precision

Specificity was investigated by comparing interferences of endogenous compounds using screening analysis of blank samples from six different sources and LLOQ samples. Acceptable criteria of LLOQ are within 80–120% of accuracy. The presence of components in plasma samples should not influence the measurement of these four analytes.

#### 3.5.4. Recovery and Matrix Effect

Recovery of D, DA, nodakenin, and DOH was obtained by measuring QC samples (at low, medium, and high concentrations) in three replicates. Recovery was performed by comparing peak area of spiked analytes after extracted blank plasma with that of analytes after extracting QC sample containing equivalent amount of analyte. Recovery of IS was carried out at working concentration (1 ng/mL) in the same manner. Recovery did not need to be 100%. However, it should be consistent, precise, and reproducible. Matrix effect was evaluated by comparing the peak area of analyte after spiking the standard solution in extracted blank plasma with that of analyte in pure standard solution containing the same amount of analyte. Matrix effect value of 100% indicated that matrix components had little influence on the detection of analytes. Relative standard deviation (RSD) value should be within ±15%.

#### 3.5.5. Stability

All stability tests including bench-top, long-term, processed sample, and freeze–thaw stability were carried out using QC samples at two different concentrations: low (1.5 ng/mL) and high (40 ng/mL). Bench-top stability test was conducted by using unextracted QC samples at room temperature for 8 h. Processed sample stability test was carried out by analyzing extracted samples in an auto sampler at 10 °C for 24 h. Freeze–thaw stability was estimated after three frozen and thawing cycles at −80 °C and room temperature on consecutive days. Long-term stability was examined after storing samples at −80 °C for a month. Stability of each coumarin was estimated by comparing the initial value with measured value after the examination (*n* = 5). The sample was considered stable if each value was less than 15% and all value of data expressed as mean ± standard deviation (mean ± SD).

#### 3.5.6. Incurred Sample Reanalysis

Incurred sample reanalysis (ISR) was carried out for 15% of total samples selected by computerized random selection. The selection criterion was set as samples were near the maximum concentration (C_max_) and elimination phase in the PK profile of the drug. A total of 17 samples were selected for ISR. Results were compared to data obtained earlier for the same sample in the same manner. Difference in percentage was calculated as absolute difference divided by the mean of the initial value and the repeat value. Acceptance criterion is: two-thirds of repeat values are 100 ± 20% of the original values.

### 3.6. Contents of D, DA, and Nodakenin in Dried Root of AGN

AGN root extract powder (0.5032 g) was accurately weighed. The powder was added into 20 mL of methanol, sonicated for 10 min, and extracted under a reflux condenser for 1 h. The extracted solution was filtered and the final volume was made up 50 mL by adding 100% methanol as test solution. Reference standard solutions of D, DA, and nodakenin were prepared by diluting each standard stock solution with methanol to final concentration range of 10–1000 µg/mL. Calibration curves were constructed using six calibration points by linear regression. A 10 µL of the test solution and reference standard solution were injected into an Alliance^®^ HPLC e2695 system equipped with 2489 UV/Vis Detector (Waters Corp., Milford, MA, USA). Contents of D, DA, and nodakenin were analyzed by the same HPLC system using Waters Nova-Pak^®^ C_18_ column (150 × 3.9 mm, 4 µm particle size, Waters, Milford, MA, USA). The mobile phase was composed of water (solution A) and acetonitrile (solution B) using gradient elution at a flow rate of 1.0 mL/min. The gradient elution program was as follows: the initial condition was maintained at 20% B for 0.5 min, linearly increased to 60% B over 5 min and held constant for up to 20.0 min, then finally returned to the initial condition (25.0 min). The wavelength used for ultraviolet detection was 330 nm.

### 3.7. Pharmacokinetic Study

Ten healthy male subjects (Korean, 23–25 years, weight of 56.7–86.2 kg, and height of 159.8–188.0 cm) were included for this clinical trial. These subjects were in good health condition prior to this study based on a physical examination, medical history, and laboratory tests. Clinical trial protocol was approved by the Institutional Review Board of Wonkwang Oriental Medicine Hospital, Gwangju, Korea (https://cris.nih.go.kr, no. KCT0001118). This clinical trial was carried out in accordance with the revised Declaration of Helsinki for biomedical research involving human subjects and Guideline for Good Clinical Practice.

All subjects fasted for at least 10 h before drug administration. They continued the fasting for another 4 h. These subjects abstained from consuming alcohol or xanthine-containing foods, or beverages during the study. A usual oral dose of AGN root extract powder was given to each subject with 240 mL of spring water. The usual dose approved by the MFDS was of 4.6 g. It contained 0.055 mg of D, 0.184 mg of DA, and 1.095 mg of nodakenin. Blood samples were taken from the forearm vein before and at 0.25, 0.5, 0.75, 1, 2, 3, 4, 6, 8, 12, and 24 h after oral administration. Samples were transferred to Vacutainer^®^ (10 mL, Becton Dickinson and Company, NJ, USA) tubes and immediately centrifuged (10,000× *g*, 10 min, 4 °C) to obtain plasma samples. Obtained plasma samples were transferred to polyethylene tubes and stored at −80 °C until analysis.

Plasma concentration–time data were analyzed with non-compartmental method using WinNonlin^®^ software (version 7.0, Pharsight^®^, Certara™ Company, Princeton, NJ, USA) to obtain pharmacokinetic parameters. The area under the plasma concentration–time curve from zero to time infinity (AUC_0–∞_) was calculated as AUC_0–t_ + C_t_/k, where C_t_ is was the last measurable concentration. AUC_0–t_ was calculated by linear trapezoidal rule from zero to the last measurable time point. Oral clearance (CL/F) was calculated as dose of each coumarin divided by AUC_0–∞_, where F was oral bioavailability. The t_1/2_ was calculated as 0.693/k, where k was terminal rate constant. All results are expressed as mean value ± standard error (mean ± SE, *n* = 10).

## 4. Conclusions

In the present study, an optimized UHPLC-MS/MS analytical method was developed and validated for simultaneous quantification of D, DA, nodakenin, and DOH as an active metabolite of D and DA in human plasma. Our results showed that this analytical method was more sensitive, cost-effective, selective, reproducible, and relatively impervious to endogenous interference than methods published in previous papers. This analytical method was successfully applied to a PK study of D, DA, nodakenin, and DOH after a usual oral dose administration of AGN root extract powder in humans. Results of this research could serve as reference for further PK studies. They also significantly contribute to quality evaluation of AGN.

## Figures and Tables

**Table 1:** Precision and accuracy of D, DA, nodakenin, and DOH in human plasma.

Added (ng/mL)	Intra-Batch (*n = 5*)			Inter-Batch (*n = 5*)		
	Measured (Mean ± SD)	Precision (CV, %)	Accuracy (%)	Measured (Mean ± SD)	Precision (CV, %)	Accuracy (%)
D						
0.05	0.0508 ± 0.00177	3.48	101.53	0.0537 ± 0.00250	4.67	107.31
0.15	0.154 ± 0.0104	6.78	102.67	0.148 ± 0.0140	9.44	98.64
8	8.00 ± 0.415	5.18	100.03	8.50 ± 0.423	4.98	106.09
40	38.3 ± 0.707	1.85	95.64	37.7 ± 0.534	1.42	94.16
DA						
0.05	0.0510 ± 0.00454	8.89	102.02	0.0497 ± 0.00231	4.65	99.34
0.15	0.159 ± 0.00607	3.83	102.67	0.156 ± 0.00638	9.44	98.64
8	7.94 ± 0.101	1.27	99.25	8.17 ± 0.479	5.86	102.09
40	40.2 ± 2.07	5.15	100.42	38.9 ± 1.35	3.46	97.36
Nodakenin						
0.05	0.0453 ± 0.00252	5.55	90.67	0.0468 ± 0.00372	7.95	93.56
0.15	0.138 ± 0.00577	4.17	92.22	0.143 ± 0.00799	5.60	95.19
8	7.60 ± 0.361	4.75	95.04	7.82 ± 0.665	8.50	97.71
40	39.6 ± 4.43	11.19	99.06	37.5 ± 1.87	4.99	93.69
DOH						
0.05	0.0523 ± 0.00475	9.09	104.58	0.0501 ± 0.00276	5.51	100.19
0.15	0.143 ± 0.00321	2.25	95.11	0.145 ± 0.0823	5.68	96.59
8	8.66 ± 0.101	1.17	108.24	8.56 ± 0.126	1.47	107.04
40	36.2 ± 1.78	4.91	90.60	38.5 ± 2.37	6.17	96.13

**Table 2:** Stability of D, DA, nodakenin, and DOH in human plasma under various storage conditions.

Compounds	Nominal Conc. (ng/mL)	Bench-Top (8 h at Room Temperature)	Processed Sample (10 °C in Auto Sampler for 24 h)	Freeze-Thaw (3 Cycles)	Long-Term (−80 °C for 30 Days)
D	0.15	101.91 ± 3.91	100.34 ± 9.38	96.54 ± 10.95	105.00 ± 9.02
	40	108.71 ± 14.16	108.94 ± 11.50	102.80 ± 12.88	102.26 ± 10.17
DA	0.15	108.58 ± 10.18	111.85 ± 12.49	103.55 ± 8.34	106.20 ± 9.09
	40	107.87 ± 4.07	103.88 ± 3.51	100.75 ± 4.12	99.35 ± 4.17
Nodakenin	0.15	101.43 ± 7.53	107.70 ± 9.77	103.49 ± 8.40	111.62 ± 8.13
	40	107.91 ± 6.81	103.17 ± 10.19	104.79 ± 12.76	101.90 ± 9.83
DOH	0.15	89.75 ± 11.39	102.43 ± 5.27	92.06 ± 2.28	87.35 ± 9.60
	40	101.81 ± 7.72	104.77 ± 2.48	104.29 ± 9.30	106.93 ± 11.89

All data were expressed as mean ± SD (*n* = 5).

**Table 3:** Pharmacokinetic parameters for D, DA, nodakenin, and DOH in humans after a usual oral dose administration of 4.6 g of AGN extract powder (mean ± SE, *n* = 10).

Parameters	D	DA	Nodakenin	DOH
C_max_ (ng/mL)	11.87 ± 1.43	7.72 ± 0.84	0.95 ± 0.17	0.92 ± 0.16
T_max_ (h)	0.44 ± 0.05	0.31 ± 0.04	0.67 ± 0.04	0.64 ± 0.06
AUC_0–∞_ (ng·h/mL)	22.33 ± 5.04	19.65 ± 3.71	3.11 ± 0.80	2.43 ± 0.45
t_1/2_ (h)	3.03 ± 0.52	4.04 ± 0.64	6.28 ± 2.47	2.62 ± 0.50
CL/F (L/h)	3.11 ± 0.42	12.00 ± 1.97	622.29 ± 159.24	-
V_d_/F (L)	11.93 ± 1.50	60.05 ± 7.30	2800.61 ± 420.12	-

**Table 4:** Optimized MRM parameters for the four coumarins.

Compounds	Precursor Ion (*m/z*)	Product ion (*m/z*)	Collision Energy (eV)	Dwell Time (msec)
D	328.8	228.8	21	200
DA	328.9	228.9	25	200
Nodakenin	409.4	248.8	13	200
DOH	246.8	212.9	37	200
Papaverine (IS)	339.8	323.8	33	200
